# Supplementary material for: Association between human herpesvirus infections and dementia or mild cognitive impairment: a systematic review protocol
Source: BMJ Open. 2017 Jun 23;7(6):e016522. doi: 10.1136/bmjopen-2017-016522 (PMC5726086; doi:10.1136/bmjopen-2017-016522)
Supplement: Supplementary Appendix 1 [file bmjopen-2017-016522supp001.pdf]

1 exp Herpes simplex
2 exp Herpes simplex virus vaccines
3 exp encephalitis, herpes simplex
4 exp Herpesvirus 1, Human
5 cold sore$.ti,ab.
6 exp Herpesvirus 2, Human
7 (genit$ herpes$ or genit$ sores).ti,ab.
8 exp Chickenpox
9 exp Chickenpox vaccine
10 exp Herpes zoster
11 exp Neuralgia, postherpetic
12 exp Herpesvirus 3, Human
13 exp Encephalitis, varicella zoster
14 (varicella or chickenpox or chicken pox or shingles or VZV or zoster).ti,ab.
15 exp Cytomegalovirus
16 exp Cytomegalovirus vaccines
17 exp Cytomegalovirus infections
18 (CMV or cytomegalovirus).ti,ab.
19 exp Herpesvirus 6, Human
20 Roseolovirus Infections/
21 Exanthema Subitum/
22 (B lymphotropic virus$ or roseola or sixth disease or exanthema subitum or exanthem criticum or Roseolovirus or pseudorubella or three?day fever).ti,ab.
23 exp Herpesvirus 7, Human
24 exp Epstein-Barr virus infections
25 exp Epstein-Barr virus
26 exp Herpesvirus 4, Human
27 (EBV or epstein-barr or burkitt adj5 lymphoma$ or glandular fever or infectious mono$ or mononucleosis or hair$ leukoplak$ or OHL).ti,ab.
28 exp Herpesvirus 8, Human
29 Sarcoma, Kaposi/
30 Lymphoma, Primary Effusion/
31 (kaposi$ sarcoma$ or Primary effusion adj2 lymphoma$ or body cavity adj2 lymphoma$).ti,ab.
32 ((HHV adj1 ("1" or "2" or "3" or "4" or "5" or "6" or "7" or "8")) or (HHV?1 or HHV?2 or HHV?3 or HHV?4 or HHV?5 or HHV?6 or HHV?7 or HHV?8)).ti,ab.
33 (HSV?1 or HSV 1 or HSV?2 or HSV 2).ti,ab.
34 herpes$.ti, ab.
35 exp acyclovir
36 ganciclovir/ or foscarnet/ or Idoxuridine/ or Trifluridine/
37 (ac?clovir or Zovirax or valac?clovir or valtrex or famc?clovir or famvir or penc?clovir or ganc?clovir or cidofovir or foscarnet$ or valganc?clovir or lubocavir or brivudin or Docosanol or Sorivudine or Idoxuridine or Trifluridine).ti,ab

38 or 1/37
39 exp dementia/
40 exp mild cognitive impairment/
41 (Alzheimer$ or dementia or Kluver-Bucy or huntington$).ti, ab.
42 (lewy$ adj2 bod$).ti, ab.
43 (Pick$ disease$ AND brain$).ti, ab.
44 ((memory or cognit$ or mental) adj5 (los$ or impair$ or deficit or problem or damage or declin$ or deteriorat$ or degenerat$ or diminish$)).ti, ab.
45 Supranuclear Palsy, Progressive/
46 (supra?nuclear pals$ or supra nuclear pals$ or PSP).ti, ab.
47 prion diseases/ or creutzfeldt-jakob syndrome/ or gerstmann-straussler-scheinker disease/ or insomnia, fatal familial/ or kuru/
48 (Prion$ disease$ or fatal familial insomnia or FFI or Gertsmann-Straussler-Scheinker syndrome or GSS or kuru or variab$ protease-sensitive prionopathy or VPSPr or transmissible spongiform encephalopath$ or TSE or Creutzfeld-Jacob$ or JCD or CJD).ti, ab.
49 or 39/49
50 38 and 51
