# Supplementary material for: Association between human herpesvirus infections and dementia or mild cognitive impairment: a systematic review protocol
Source: BMJ Open. 2017 Jun 23;7(6):e016522. doi: 10.1136/bmjopen-2017-016522 (PMC5726086; doi:10.1136/bmjopen-2017-016522)
Supplement: Author's manuscript [file bmjopen-2017-016522.draft_revisions.pdf]

BMJ Open

Association between human herpesvirus infections and dementia onset: a systematic review protocol

Journal:	*BMJ Open*
Manuscript ID	bmjopen-2017-016522
Article Type:	Protocol
Date Submitted by the Author:	20-Feb-2017
Complete List of Authors:	Warren-Gash, Charlotte; London School of Hygiene and Tropical Medicine, Epidemiology and Population Health<br>Forbes, Harriet; London School of Hygiene and Tropical Medicine Faculty of Epidemiology and Population Health, NCDE<br>Breuer, Judith; University College London<br>Hayward, Andrew; University College London, Infection and Population Health<br>Mavrodaris, Angelique ; MRC Epidemiology Unit<br>Ridha, Basil; University College London Institute of Neurology<br>Rossor, Martin; National Hospital for Neurology and Neurosurgery, Dementia Research Centre<br>Thomas, Sara; London School of Hygiene & Tropical Medicine, Faculty of Epidemiology & Population Health<br>Smeeth, Liam; London School of Hygiene and Tropical Medicine, Epidemiology and Population Health
**Primary Subject Heading**:	Neurology
Secondary Subject Heading:	Infectious diseases, Epidemiology
Keywords:	Dementia < NEUROLOGY, Human herpesviruses, Systematic review protocol

SCHOLARONE™ Manuscripts

For peer review only - http://bmjopen.bmj.com/site/about/guidelines.xhtml

Association between human herpesvirus infections and dementia onset: a systematic review protocol

Charlotte Warren-Gash[1], Harriet Forbes[1], Judy Breuer[2], Andrew C. Hayward[3], Angelique Mavrodaris[4], Basil H. Ridha[5], Martin Rossor[5,6], Sara Thomas[1], Liam Smeeth[1]

Affiliations:

1. Faculty of Epidemiology & Population Health, London School of Hygiene & Tropical Medicine, Keppel Street, London, United Kingdom
2. Division of Infection & Immunity, University College London, Gower Street, London, United Kingdom
3. Institute of Epidemiology and Health Care, University College London, 222 Euston Road, London, United Kingdom
4. Cambridge Institute of Public Health, Forvie Site, Robinson Way, Cambridge, United Kingdom
5. NIHR Queen Square Dementia Biomedical Research Unit, University College London, Queen Square, London
6. Dementia Research Centre, Institute of Neurology, University College London, Queen Square, London, United Kingdom

Correspondence to:

Dr Charlotte Warren-Gash. Email: charlotte.warren-gash1@lshtm.ac.uk, tel: 020 7927 2796

Word count: 1884 excluding abstract and references

For peer review only

For peer review only - http://bmjopen.bmj.com/site/about/guidelines.xhtml

ABSTRACT (300 words)

Introduction: Persisting neurotropic viruses are proposed to increase the risk of dementia but evidence of association from robust, adequately powered population studies is lacking. This is essential to inform clinical trials of targeted preventive interventions.

Methods and analysis: We will carry out a comprehensive systematic review of published and grey literature of the association between infection with, reactivation of, vaccination against or treatment of any of the eight human herpesviruses AND dementia or mild cognitive impairment (MCI). We will search the Cochrane Library, Embase, Global Health, Medline, PsychInfo, Scopus, Web of Science, clinical trials registers, the New York Academy of Medicine Grey Literature Report, Electronic Theses Online Service through the British Library and the ISI Conference Proceedings Citation Index for RCTs, cohort, case control, case crossover or self-controlled case series studies reported in any language up to January 2017. Titles, abstracts and full-text screening will be conducted by two researchers independently. Data will be extracted systematically from eligible studies using a piloted template. We will assess risk of bias of individual studies in line with the Cochrane Collaboration tool. We will conduct a narrative synthesis, grouping studies by exposure and outcome definitions and will describe any differences by population subgroups and dementia subtypes. We will consider performing meta-analyses if there are adequate numbers of sufficiently homogeneous studies. The overall quality of cumulative evidence will be assessed using selected Grading of Recommendations, Assessment, Development and Evaluations (GRADE) criteria.

Ethics and Dissemination: As this is a review of existing studies, no ethical approval is required. Results will be disseminated through a peer-reviewed publication and at national and international conferences. We anticipate the review will clarify the current extent and quality of evidence for a link between herpesviruses and dementia, identify gaps and inform the direction of future research.

Prospero registration number: CRD42017054684

For peer review only

For peer review only - http://bmjopen.bmj.com/site/about/guidelines.xhtml

Strengths and limitations of this study

- This systematic review will comprehensively evaluate published and grey literature on the association between infection with, reactivation of, vaccination against or treatment of any of the eight human herpesviruses and dementia or mild cognitive impairment.
- One key strength of our review is that it will assess the evidence for differential effects of human herpesviruses on dementia risk in different population subgroups, for example, by age, ethnicity and immune status to inform the design and targeting of randomised controlled trials of preventive interventions to reduce dementia risk.
- A lack of adequately powered studies or heterogeneity of studies selected for inclusion in terms of study design, exposure and outcome definitions or study population may restrict our capacity to derive clear conclusions about the study question.

For peer review only

For peer review only - http://bmjopen.bmj.com/site/about/guidelines.xhtml

INTRODUCTION

Rationale

Dementia has now overtaken ischaemic heart disease as the leading cause of death in England and Wales[1]. The prevalence is forecast to rise rapidly as the population ages, with the number of dementia cases worldwide projected to reach 131.5 million in 2050 compared to 46.8 million in 2015[2], assuming that age-specific prevalence rates remain stable[3]. Only one third of cases are attributable to potentially preventable risk factors[4] and there are no effective treatments. It is therefore essential to identify other modifiable determinants of dementia.

Viral aetiologies for dementia have been postulated for decades, but population level evidence is inconsistent and no antiviral strategies to reduce dementia risk are in routine clinical use. Recent interest has focussed on herpes simplex virus type 1 (HSV1) – one of eight human herpesviruses that routinely infect humans. After primary infection, all human herpesviruses establish latency within specific tissues, with the alpha herpesviruses HSV1, HSV2 and varicella zoster virus (VZV) demonstrating neurotropism through their characteristic persistence in sensory nerve ganglia[5].

HSV1 infection of neuronal and glial cell cultures induces cellular changes similar to those seen in Alzheimer's disease (AD)[6-8], and which are reversible using antiviral agents[9]. Post-mortem studies show that HSV establishes latency in human brain tissue: viral genomic sequences have been detected in up to 35% of neurologically asymptomatic individuals[10]; HSV reactivation from latency occurs in explanted mouse brainstems[11,12]. A meta-analysis of mainly small case control studies of varying quality published in 2015 suggested that HSV1 seropositivity was associated with an increased risk of AD (pooled OR 1.38 (95% C.I. 1.03-1.84)) as was the presence of Epstein-Barr virus (EBV) antibodies (pooled OR 1.55 (95% C.I. 1.12-2.13))[13]. Although the presence of any of six herpesviruses in brain tissue was associated with AD in case control studies (pooled OR 1.38 (95% C.I. 1.14-1.66)), the temporal sequence and clinical significance of this finding is unclear.

For peer review only - http://bmjopen.bmj.com/site/about/guidelines.xhtml

For peer review only

Our study will have some key differences to this previous review: we will consider the effect of infection with and reactivation of all eight human herpesviruses on both AD and other neurodegenerative diseases causing dementia or MCI; we will investigate whether preventing or treating herpesvirus infections affects dementia risk; we will assess the evidence for differential effects of human herpesviruses on dementia risk in different population subgroups e.g. by age, ethnicity, socio-economic status and immune status to inform the design and targeting of randomised controlled trials of preventive interventions.

For peer review only

Objectives

The overall aim is to determine whether people infected with any of the eight human herpesviruses are at greater risk of subsequently developing dementia or MCI than those without evidence of herpesvirus infection.

Specific research questions include:

1. Does primary infection with or reactivation of human herpesviruses affect the risk of dementia or MCI?
2. Does preventing or treating human herpesviruses modulate dementia or MCI risk?
3. Does any association between human herpesviruses and dementia or MCI vary by population subgroups or between subtypes of dementia?

METHODS

This systematic review protocol has been prepared according to the PRISMA-P (Preferred Reporting Items for Systematic Review and Meta-Analysis Protocols) 2015 statement[14].

Eligibility criteria

For peer review only - http://bmjopen.bmj.com/site/about/guidelines.xhtml

Study design: We will include all randomised controlled trials, prospective and retrospective cohort studies, case control studies, self-controlled case series and case crossover studies that either present an estimate of effect or provide sufficient data for an effect estimate to be calculated.

Population: Our study population will include adults aged 18 years and over, with results stratified where possible by age group and immune status. Studies conducted in any setting e.g. hospital in-patient, out-patient, primary care or the community will be considered. There will be no language or geographic limits. No animal studies will be included.

Exposure: Our exposures are infection with or reactivation of any of the eight human herpesvirus infections (defined either clinically or through appropriate laboratory criteria), herpesvirus vaccinations (e.g. zostavax) and herpesvirus treatments (e.g. with antiviral agents such as acyclovir).

Comparators: Comparators will vary by study design and will include subjects randomised not to receive an antiviral intervention or vaccination (RCTs), people unexposed to herpesvirus infections (cohort studies and case control studies) and person time unexposed to herpesviruses (self-controlled case series or case crossover studies).

Outcome: Primary outcomes are dementia (all types), diagnosed either clinically, with or without neuroimaging or by histopathology, and MCI, characterised clinically. Where possible, dementia will be further categorised by type, e.g. AD, vascular dementia, fronto-temporal dementia and its sub-categories, Lewy body dementia, and other rare subtypes including Huntington's disease and prion diseases.

Literature searches

We will search the Cochrane Library, Embase, Global Health, Medline, PsychInfo, Scopus and Web of Science from dates of inception to January 2017. We will also search clinical trials registers and grey literature including ClinicalTrials.gov, the New York Academy of Medicine Grey Literature Report (www.greylit.org), the Electronic Theses Online Service through the British Library (http://ethos.bl.uk) and the ISI Conference Proceedings Citation Index (http://isknowledge.com). We

For peer review only - http://bmjopen.bmj.com/site/about/guidelines.xhtml

For peer review only

will use medical subject heading (MeSH) terms and keyword searches (in the title and abstract) to capture studies of infection with, vaccination against or antiviral treatment of any of the eight human herpesviruses (HSV1, HSV2, VZV, EBV, cytomegalovirus (CMV), human herpesvirus 6 (HHV6), HHV7, HHV8) AND dementia (including subtypes) or MCI. This strategy will be supplemented by reviewing reference lists of eligible articles and relevant reviews. The search strategy has been developed in Medline and will be translated for use in other databases (Appendix 1).

Study records

Data management: Search results will be uploaded into an EndNote database (V7.5) and de-duplicated.

Selection process: All titles and abstracts will be scanned for eligibility by two researchers in parallel. The full texts of all articles that potentially meet inclusion criteria will then be obtained and again reviewed in parallel. We will note reasons for rejection of articles at this stage according to a hierarchical list (ineligible study design, wrong exposure, wrong outcome, insufficient information to calculate an effect estimate). Any discrepancies will be discussed between reviewers and consultation with a third reviewer will be carried out where necessary.

Data collection process: The data items listed below will be incorporated into a pilot data extraction table. Parallel data extraction will be carried out for the first three included studies by two reviewers and changes to the extraction table made as required. Any discrepancies will be discussed, and resolved through consultation with a third reviewer if necessary. Data will be extracted for each remaining study by a single reviewer. We will also consider contacting corresponding authors of published studies to obtain any further information needed using a standardised email template.

Data items

We will design a data extraction table to collect information on the following domains:

For peer review only - http://bmjopen.bmj.com/site/about/guidelines.xhtml

Population: characteristics of the study population (e.g. sex and age distribution, ethnicity, immune status), recruitment and sampling methods, inclusion/exclusion criteria;

Exposure: exposure status definition and identification, number of exposed subjects, any exclusions;

Comparators: identification and definition of unexposed individuals, number of unexposed subjects, any exclusions;

Outcomes: definition and identification of primary (dementia or MCI) and secondary outcomes (dementia subtypes), number of subjects, any exclusions;

Study characteristics: authors, publication year, setting/source of participants, design, period of study, length of follow-up time (if relevant), aims and objectives.

We will record both unadjusted and fully adjusted effect estimates for the association between each of the human herpesviruses and risk or rate of dementia or MCI. Details of the confounders measured and adjusted for will be noted. Results of any additional stratified analyses will also be recorded, e.g. on virus effects in different population subgroups or on dementia subtypes, including categories of severity.

Outcomes and prioritization

The primary outcomes are dementia (all types) and MCI. Where possible, the precise diagnosis of the condition leading to dementia or MCI will be recorded, e.g. AD, vascular dementia, fronto-temporal dementia and its sub-categories, Lewy body dementia, and other rare subtypes including Huntington's disease and prion diseases. A priori, we anticipate that most studies will report on AD. We have, however, opted for a broader primary outcome definition: excluding studies that do not record dementia type will limit the statistical power to detect an effect yet identifying a precise dementia aetiology is challenging, especially when histopathological diagnosis is not available. We will prioritise studies in which herpesvirus status was ascertained prior to the occurrence of an outcome event when assessing study quality. These are likely to be longitudinal prospective studies

For peer review only

that generate incidence rate ratios or hazard ratios, but we will also consider estimates of odds ratios from case control studies.

Risk of bias in individual studies

We will assess the risk of bias in individual studies in line with the Cochrane Collaboration approach[15]. For RCTs this will include consideration of the effects of selection bias, performance bias, detection bias, attrition bias and reporting bias. For observational studies we will consider relevant domains including selection of participants, measurement of variables, control for confounding and missing data. For each study, each component will be assigned a risk of bias category 'high risk', 'low risk' or 'unclear risk' and a summary risk of bias table produced. Two reviewers will assign risk of bias categories in parallel for the first three studies and, as before, any discrepancies that cannot be resolved will be discussed with a third reviewer. One reviewer will then assign risk of bias categories for the remaining studies.

Data synthesis

We will conduct a narrative synthesis in which evidence is grouped by exposure and outcome definitions and we will additionally summarise the results in tables. We will assess whether there are sufficient data to investigate the effect of herpesviruses on population subgroups or subtypes of dementia. We will investigate whether vaccination to prevent herpesvirus infection or reactivation e.g. using chickenpox vaccine or herpes zoster vaccine mitigates the risk of dementia. We will also present any evidence for a modulating effect of antiviral treatment against herpesviruses on dementia.

We will consider performing a meta-analysis if there are sufficient numbers of studies that are homogeneous in study design, population and outcomes to calculate a pooled effect estimate. The choice of fixed or random effects model will be guided by the level of statistical heterogeneity

For peer review only - http://bmjopen.bmj.com/site/about/guidelines.xhtml

(assessed using the I^2 statistic). Sources of heterogeneity will be explored by first, removing studies at high risk of bias and second, considering the use of meta-regression to compare summary estimates from different study-level characteristics, such as age of the study population, study design and dementia or MCI diagnosis. If there are sufficient numbers of studies, we will investigate the risk of publication bias using a funnel plot. All of the statistical analysis will be performed using STATA version 14.0.

Cumulative evidence

The quality of cumulative evidence across studies for an association of each herpesvirus with each outcome will then be assessed using selected GRADE criteria that are relevant to the included studies[16]. As well as risk of bias these include inconsistency, indirectness, imprecision, publication bias and any additional domains deemed appropriate to categorise the strength of evidence as 'high', 'moderate' or 'low/very low'.

ETHICS AND DISSEMINATION

Ethical approval was not required as this study is a systematic review of previously published studies. The protocol was registered with the International Prospective Register of Systematic Reviews (PROSPERO) on 7 January 2017 (Registration number: CRD42017054684). Any future amendments will be documented on the PROSPERO website. Results will be submitted for publication in a peer-reviewed journal and presented at national and international conferences.

Contributors

CWG conceived and designed the study, drafted and revised the protocol following author comments; HF contributed to the design of the study and revised the paper critically; JB contributed to the conception and design of the study and revised the paper critically; ACH contributed to the conception and design of the study and revised the paper critically; AM contributed to the design of

For peer review only - http://bmjopen.bmj.com/site/about/guidelines.xhtml

the study and revised the paper critically; BHR contributed to the design of the study and revised the paper critically; MR contributed to the design of the study and revised the paper critically; SLT contributed to the design of the study and revised the paper critically; LS contributed to the conception and design of the study and revised the paper critically. All authors approved the final version of the protocol.

Funding

This work was supported by a Wellcome Intermediate Clinical Fellowship awarded to CW-G (201440/Z/16/Z). LS is supported by a Senior Clinical Fellowship from Wellcome (098504/Z/12/Z). JB receives funding from the NIHR UCL/UCLH BRC. The funder had no direct role in developing the protocol.

Competing interests

All authors report no conflicts of interest

References

1. Office for National Statistics. Deaths registered in England and Wales (Series DR): 2015. Release date 14 Nov 2016. Available from: https://www.ons.gov.uk/peoplepopulationandcommunity/birthsdeathsandmarriages/deaths/bulletins/deathsregisteredinenglandandwalesseriesdr/2015. Last accessed 13 Feb 2017.

2. Prince M, Wimo A, Guerchet M, Ali G-C, Wu Y-T, Prina M. World Alzheimer Report 2015. The Global Impact of Dementia: An Analysis of Prevalence, Incidence, Cost and Trends. London, UK: Alzheimer's Disease International. Aug 2015. Available from: https://www.alz.co.uk/research/WorldAlzheimerReport2015.pdf Last accessed 13 Feb 2017.

3. Prince M, Ali G-C, Guerchet M, Prina AM, Albanese E, Wu Y-T. Recent global trends in the prevalence and incidence of dementia, and survival with dementia. Alzheimers Res Ther. 2016; 8(1): 23.

4. Norton S, Matthews FE, Barnes DE, Yaffe K, Brayne C. Potential for primary prevention of Alzheimer's disease: an analysis of population-based data. Lancet Neurol. 2014; 13(8): 788–794.

5. Whitley RJ. Herpesviruses. In: Baron S, editor. Medical Microbiology. 4th ed. Galveston (TX): University of Texas Medical Branch at Galveston; 1996. Available from: http://www.ncbi.nlm.nih.gov/books/NBK8157/ Last accessed 13 Feb 2017

For peer review only - http://bmjopen.bmj.com/site/about/guidelines.xhtml

6. Alvarez G, Aldudo J, Alonso M, Santana S, Valdivieso F. Herpes simplex virus type 1 induces nuclear accumulation of hyperphosphorylated tau in neuronal cells. J Neurosci Res. 2012; 90(5): 1020–9.

7. Wozniak MA, Itzhaki RF, Shipley SJ, Dobson CB. Herpes simplex virus infection causes cellular β-amyloid accumulation and secretase upregulation. Neurosci Lett. 2007; 429(2–3): 95–100.

8. De Chiara G, Marcocci ME, Civitelli L, Argnani R, Piacentini R, Ripoli C, et al. APP Processing Induced by Herpes Simplex Virus Type 1 (HSV-1) Yields Several APP Fragments in Human and Rat Neuronal Cells. Blaho JA, editor. PLoS ONE. 2010; 5(11): e13989.

9. Wozniak MA, Frost AL, Preston CM, Itzhaki RF. Antivirals reduce the formation of key Alzheimer’s disease molecules in cell cultures acutely infected with herpes simplex virus type 1. PloS One. 2011; 6(10): e25152.

10. Baringer JR, Pisani P. Herpes simplex virus genomes in human nervous system tissue analyzed by polymerase chain reaction. Ann Neurol. 1994; 36(6): 823–9.

11. Yao H-W, Ling P, Chen S-H, Tung Y-Y, Chen S-H. Factors affecting herpes simplex virus reactivation from the explanted mouse brain. Virology. 2012; 433(1): 116–23.

12. Chen S-H, Yao H-W, Huang W-Y, Hsu K-S, Lei H-Y, Shiau A-L, et al. Efficient Reactivation of Latent Herpes Simplex Virus from Mouse Central Nervous System Tissues. J Virol. 2006; 80(24): 12387–92.

13. Steel AJ, Eslick GD. Herpes Viruses Increase the Risk of Alzheimer’s Disease: A Meta-Analysis. J Alzheimers Dis. 2015; 47(2): 351–64.

14. Moher D, Shamseer L, Clarke M, Ghersi D, Liberati A, Petticrew M, et al. Preferred reporting items for systematic review and meta-analysis protocols (PRISMA-P) 2015 statement. Syst Rev. 2015; 4(1):1.

15. Higgins JPT, Altman DG, Gøtzsche PC, Jüni P, Moher D, Oxman AD, et al. The Cochrane Collaboration’s tool for assessing risk of bias in randomised trials. BMJ. 2011; 343: d5928.

16. Atkins D, Best D, Briss PA, Eccles M, Falck-Ytter Y, Flottorp S, et al. Grading quality of evidence and strength of recommendations. BMJ. 2004; 328(7454): 1490.

For peer review only

For peer review only - http://bmjopen.bmj.com/site/about/guidelines.xhtml

1 exp Herpes simplex

2 exp Herpes simplex virus vaccines

3 exp encephalitis, herpes simplex

4 exp Herpesvirus 1, Human

5 cold sore$.ti,ab.

6 exp Herpesvirus 2, Human

7 (genit$ herpes$ or genit$ sores).ti,ab.

8 exp Chickenpox

9 exp Chickenpox vaccine

10 exp Herpes zoster

11 exp Neuralgia, postherpetic

12 exp Herpesvirus 3, Human

13 exp Encephalitis, varicella zoster

14 (varicella or chickenpox or chicken pox or shingles or VZV or zoster).ti,ab.

15 exp Cytomegalovirus

16 exp Cytomegalovirus vaccines

17 exp Cytomegalovirus infections

18 (CMV or cytomegalovirus).ti,ab.

19 exp Herpesvirus 6, Human

20 Roseolovirus Infections/

21 Exanthema Subitum/

22 (B lymphotropic virus$ or roseola or sixth disease or exanthema subitum or exanthem criticum or Roseolovirus or pseudorubella or three?day fever).ti,ab.

23 exp Herpesvirus 7, Human

24 exp Epstein-Barr virus infections

25 exp Epstein-Barr virus

26 exp Herpesvirus 4, Human

27 (EBV or epstein-barr or burkitt adj5 lymphoma$ or glandular fever or infectious mono$ or mononucleosis or hair$ leukoplak$ or OHL).ti,ab.

28 exp Herpesvirus 8, Human

29 Sarcoma, Kaposi/

30 Lymphoma, Primary Effusion/

31 (kaposi$ sarcoma$ or Primary effusion adj2 lymphoma$ or body cavity adj2 lymphoma$).ti,ab.

32 ((HHV adj1 ("1" or "2" or "3" or "4" or "5" or "6" or "7" or "8")) or (HHV?1 or HHV?2 or HHV?3 or HHV?4 or HHV?5 or HHV?6 or HHV?7 or HHV?8)).ti,ab.

33 (HSV?1 or HSV 1 or HSV?2 or HSV 2).ti,ab.

34 herpes$.ti, ab.

35 exp acyclovir

36 ganciclovir/ or foscarnet/ or Idoxuridine/ or Trifluridine/

37 (ac?clovir or Zovirax or valac?clovir or valtrex or famc?clovir or famvir or penc?clovir or ganc?clovir or cidofovir or foscarnet$ or valganc?clovir or lubocavir or brivudin or Docosanol or Sorivudine or Idoxuridine or Trifluridine).ti,ab

38 or 1/37

39 exp dementia/

40 exp mild cognitive impairment/

41 (Alzheimer$ or dementia or Kluver-Bucy or huntington$).ti, ab.

42 (lewy$ adj2 bod$).ti, ab.

43 (Pick$ disease$ AND brain$).ti, ab.

44 ((memory or cognit$ or mental) adj5 (los$ or impair$ or deficit or problem or damage or declin$ or deteriorat$ or degenerat$ or diminish$)).ti, ab.

45 Supranuclear Palsy, Progressive/

46 (supra?nuclear pals$ or supra nuclear pals$ or PSP).ti, ab.

47 prion diseases/ or creutzfeldt-jakob syndrome/ or gerstmann-straussler-scheinker disease/ or insomnia, fatal familial/ or kuru/

48 (Prion$ disease$ or fatal familial insomnia or FFI or Gertsmann-Straussler-Scheinker syndrome or GSS or kuru or variab$ protease-sensitive prionopathy or VPSPr or transmissible spongiform encephalopath$ or TSE or Creutzfeld-Jacob$ or JCD or CJD).ti, ab.

49 or 39/49

50 38 and 51

For peer review only

PRISMA-P (Preferred Reporting Items for Systematic review and Meta-Analysis Protocols) 2015 checklist: recommended items to address in a systematic review protocol*

Section and topic	Item No	Checklist item		
**ADMINISTRATIVE INFORMATION**				
Title:				
Identification	1a	Identify the report as a protocol of a systematic review	✓	P1
Update	1b	If the protocol is for an update of a previous systematic review, identify as such	-	-
Registration	2	If registered, provide the name of the registry (such as PROSPERO) and registration number	✓	P2
Authors:				
Contact	3a	Provide name, institutional affiliation, e-mail address of all protocol authors; provide physical mailing address of corresponding author	✓	P1
Contributions	3b	Describe contributions of protocol authors and identify the guarantor of the review	✓	P10/11
Amendments	4	If the protocol represents an amendment of a previously completed or published protocol, identify as such and list changes; otherwise, state plan for documenting important protocol amendments	-	-
Support:				
Sources	5a	Indicate sources of financial or other support for the review	✓	P11
Sponsor	5b	Provide name for the review funder and/or sponsor	✓	P11
Role of sponsor or funder	5c	Describe roles of funder(s), sponsor(s), and/or institution(s), if any, in developing the protocol	✓	P11
**INTRODUCTION**				
Rationale	6	Describe the rationale for the review in the context of what is already known	✓	P4/5
Objectives	7	Provide an explicit statement of the question(s) the review will address with reference to participants, interventions, comparators, and outcomes (PICO)	✓	P5
**METHODS**				
Eligibility criteria	8	Specify the study characteristics (such as PICO, study design, setting, time frame) and report characteristics (such as years considered, language, publication status) to be used as criteria for eligibility for the review	✓	P6
Information sources	9	Describe all intended information sources (such as electronic databases, contact with study authors, trial registers or other grey literature sources) with planned dates of coverage	✓	P6/7

For peer review only

Search strategy	10	Present draft of search strategy to be used for at least one electronic database, including planned limits, such that it could be repeated	✓	P7 (and in an appendices)
Study records:				
Data management	11a	Describe the mechanism(s) that will be used to manage records and data throughout the review	✓	P7
Selection process	11b	State the process that will be used for selecting studies (such as two independent reviewers) through each phase of the review (that is, screening, eligibility and inclusion in meta-analysis)	✓	P7
Data collection process	11c	Describe planned method of extracting data from reports (such as piloting forms, done independently, in duplicate), any processes for obtaining and confirming data from investigators	✓	P7
Data items	12	List and define all variables for which data will be sought (such as PICO items, funding sources), any pre-planned data assumptions and simplifications	✓	P7/8
Outcomes and prioritization	13	List and define all outcomes for which data will be sought, including prioritization of main and additional outcomes, with rationale	✓	P8
Risk of bias in individual studies	14	Describe anticipated methods for assessing risk of bias of individual studies, including whether this will be done at the outcome or study level, or both; state how this information will be used in data synthesis	✓	P9
Data synthesis	15a	Describe criteria under which study data will be quantitatively synthesised	✓	P9/10
	15b	If data are appropriate for quantitative synthesis, describe planned summary measures, methods of handling data and methods of combining data from studies, including any planned exploration of consistency (such as I^2, Kendall's τ)	✓	P9/10
	15c	Describe any proposed additional analyses (such as sensitivity or subgroup analyses, meta-regression)	✓	P9/10
	15d	If quantitative synthesis is not appropriate, describe the type of summary planned	✓	P9/10
Meta-bias(es)	16	Specify any planned assessment of meta-bias(es) (such as publication bias across studies, selective reporting within studies)	✓	P9/10
Confidence in cumulative evidence	17	Describe how the strength of the body of evidence will be assessed (such as GRADE)	✓	P10

*** It is strongly recommended that this checklist be read in conjunction with the PRISMA-P Explanation and Elaboration (cite when available) for important clarification on the items. Amendments to a review protocol should be tracked and dated.** **The copyright for PRISMA-P (including checklist) is held by the PRISMA-P Group and is distributed under a Creative Commons Attribution Licence 4.0.**

From: Shamseer L, Moher D, Clarke M, Ghersi D, Liberati A, Petticrew M, Shekelle P, Stewart L, PRISMA-P Group. Preferred reporting items for systematic review and meta-analysis protocols (PRISMA-P) 2015: elaboration and explanation. BMJ. 2015 Jan 2;349(jan02 1):g7647.

For peer review only

BMJ Open

Association between human herpesvirus infections and dementia or mild cognitive impairment: a systematic review protocol

Journal:	*BMJ Open*
Manuscript ID	bmjopen-2017-016522.R1
Article Type:	Protocol
Date Submitted by the Author:	15-May-2017
Complete List of Authors:	Warren-Gash, Charlotte; London School of Hygiene and Tropical Medicine, Epidemiology and Population Health<br>Forbes, Harriet; London School of Hygiene and Tropical Medicine Faculty of Epidemiology and Population Health, NCDE<br>Breuer, Judith; University College London<br>Hayward, Andrew; University College London, Infection and Population Health<br>Mavrodaris, Angelique ; MRC Epidemiology Unit<br>Ridha, Basil; University College London Institute of Neurology<br>Rossor, Martin; National Hospital for Neurology and Neurosurgery, Dementia Research Centre<br>Thomas, Sara; London School of Hygiene & Tropical Medicine, Faculty of Epidemiology & Population Health<br>Smeeth, Liam; London School of Hygiene and Tropical Medicine, Epidemiology and Population Health
**Primary Subject Heading**:	Neurology
Secondary Subject Heading:	Infectious diseases, Epidemiology
Keywords:	Dementia < NEUROLOGY, Human herpesviruses, Systematic review protocol

SCHOLARONE™
Manuscripts

Association between human herpesvirus infections and dementia or mild cognitive impairment: a systematic review protocol

Charlotte Warren-Gash[1], Harriet Forbes[1], Judy Breuer[2], Andrew C. Hayward[3], Angelique Mavrodaris[4], Basil H. Ridha[5], Martin Rossor[5,6], Sara L. Thomas[1], Liam Smeeth[1]

Affiliations:

1. Faculty of Epidemiology & Population Health, London School of Hygiene & Tropical Medicine, Keppel Street, London, United Kingdom
2. Division of Infection & Immunity, University College London, Gower Street, London, United Kingdom
3. Institute of Epidemiology and Health Care, University College London, 222 Euston Road, London, United Kingdom
4. Cambridge Institute of Public Health, Forvie Site, Robinson Way, Cambridge, United Kingdom
5. NIHR Queen Square Dementia Biomedical Research Unit, University College London, Queen Square, London
6. Dementia Research Centre, Institute of Neurology, University College London, Queen Square, London, United Kingdom

Correspondence to:

Dr Charlotte Warren-Gash. Email: charlotte.warren-gash1@lshtm.ac.uk, tel: 020 7927 2796

Word count: 1977 excluding abstract and references

For peer review only

For peer review only - http://bmjopen.bmj.com/site/about/guidelines.xhtml

ABSTRACT (300 words)

Introduction: Persisting neurotropic viruses are proposed to increase the risk of dementia but evidence of association from robust, adequately powered population studies is lacking. This is essential to inform clinical trials of targeted preventive interventions.

Methods and analysis: We will carry out a comprehensive systematic review of published and grey literature of the association between infection with, reactivation of, vaccination against or treatment of any of the eight human herpesviruses AND dementia or mild cognitive impairment (MCI). We will search the Cochrane Library, Embase, Global Health, Medline, PsychInfo, Scopus, Web of Science, clinical trials registers, the New York Academy of Medicine Grey Literature Report, Electronic Theses Online Service through the British Library and the ISI Conference Proceedings Citation Index for RCTs, cohort, case control, case crossover or self-controlled case series studies reported in any language up to January 2017. Titles, abstracts and full-text screening will be conducted by two researchers independently. Data will be extracted systematically from eligible studies using a piloted template. We will assess risk of bias of individual studies in line with the Cochrane Collaboration tool. We will conduct a narrative synthesis, grouping studies by exposure and outcome definitions and will describe any differences by population subgroups and dementia subtypes. We will consider performing meta-analyses if there are adequate numbers of sufficiently homogeneous studies. The overall quality of cumulative evidence will be assessed using selected Grading of Recommendations, Assessment, Development and Evaluations (GRADE) criteria.

Ethics and Dissemination: As this is a review of existing studies, no ethical approval is required. Results will be disseminated through a peer-reviewed publication and at national and international conferences. We anticipate the review will clarify the current extent and quality of evidence for a link between herpesviruses and dementia, identify gaps and inform the direction of future research.

Prospero registration number: CRD42017054684

For peer review only

For peer review only - http://bmjopen.bmj.com/site/about/guidelines.xhtml

Strengths and limitations of this study

- This systematic review will comprehensively evaluate published and grey literature on the association between infection with, reactivation of, vaccination against or treatment of any of the eight human herpesviruses and dementia or mild cognitive impairment.
- One key strength of our review is that it will assess the evidence for differential effects of human herpesviruses on dementia risk in different population subgroups, for example, by age, ethnicity and immune status to inform the design and targeting of randomised controlled trials of preventive interventions to reduce dementia risk.
- A lack of adequately powered studies or heterogeneity of studies selected for inclusion in terms of study design, exposure and outcome definitions or study population may restrict our capacity to derive clear conclusions about the study question.

For peer review only

For peer review only - http://bmjopen.bmj.com/site/about/guidelines.xhtml

INTRODUCTION

Rationale

Dementia has now overtaken ischaemic heart disease as the leading cause of death in England and Wales[1]. The prevalence is forecast to rise rapidly as the population ages, with the number of dementia cases worldwide projected to reach 131.5 million in 2050 compared to 46.8 million in 2015[2], assuming that age-specific prevalence rates remain stable[3]. After accounting for overlap between risk factors, only around one third of dementia cases are attributable to potentially preventable factors[4] and there are no effective treatments. It is therefore essential to identify other modifiable determinants.

Viral aetiologies for dementia have been postulated for decades, but population level evidence is inconsistent and no antiviral strategies to reduce dementia risk are in routine clinical use. Recent interest has focussed on herpes simplex virus type 1 (HSV1)[5,6] – one of eight human herpesviruses that routinely infect humans. After primary infection, all human herpesviruses establish latency within specific tissues, with the alpha herpesviruses HSV1, HSV2 and varicella zoster virus (VZV) demonstrating neurotropism through their characteristic persistence in sensory nerve ganglia[7].

HSV1 infection of neuronal and glial cell cultures induces cellular changes similar to those seen in Alzheimer's disease (AD)[8–10], and which are reversible using antiviral agents[11]. Post-mortem studies show that HSV establishes latency in human brain tissue: viral genomic sequences have been detected in up to 35% of neurologically asymptomatic individuals[12]; HSV reactivation from latency occurs in explanted mouse brainstems[13,14]. A meta-analysis of mainly small case control studies of varying quality published in 2015 suggested that HSV1 seropositivity was associated with an increased risk of AD (pooled OR 1.38 (95% C.I. 1.03-1.84)) as was the presence of Epstein-Barr virus (EBV) antibodies (pooled OR 1.55 (95% C.I. 1.12-2.13))[15]. Although the presence of any of six

For peer review only - http://bmjopen.bmj.com/site/about/guidelines.xhtml

herpesviruses in brain tissue was associated with AD in case control studies (pooled OR 1.38 (95% C.I. 1.14-1.66)), the temporal sequence and clinical significance of this finding is unclear.

Our study will have some key differences to this previous review: we will consider the effect of infection with and reactivation of all eight human herpesviruses on both AD and other neurodegenerative diseases causing dementia or MCI; we will investigate whether preventing or treating herpesvirus infections affects dementia risk; we will assess the evidence for differential effects of human herpesviruses on dementia risk in different population subgroups e.g. by age, ethnicity, socio-economic status and immune status to inform the design and targeting of randomised controlled trials of preventive interventions.

Objectives

The overall aim is to determine whether people infected with any of the eight human herpesviruses are at greater risk of subsequently developing dementia or MCI than those without evidence of herpesvirus infection.

Specific research questions include:

1. Does primary infection with or reactivation of human herpesviruses affect the risk of dementia or MCI?
2. Does preventing or treating human herpesviruses modulate dementia or MCI risk?
3. Does any association between human herpesviruses and dementia or MCI vary by population subgroups or between subtypes of dementia?

METHODS

This systematic review protocol has been prepared according to the PRISMA-P (Preferred Reporting Items for Systematic Review and Meta-Analysis Protocols) 2015 statement[16].

For peer review only

Eligibility criteria

Study design: We will include all randomised controlled trials, prospective and retrospective cohort studies, case control studies, self-controlled case series and case crossover studies that either present an estimate of effect or provide sufficient data for an effect estimate to be calculated.

Population: Our study population will include adults aged 18 years and over, with results stratified where possible by age group, immune status and APOE4 status. Studies conducted in any setting e.g. hospital in-patient, out-patient, primary care or the community will be considered. There will be no language or geographic limits. No animal studies will be included.

Exposure: Our exposures are infection with or reactivation of any of the eight human herpesvirus infections (defined either clinically or through appropriate laboratory criteria), herpesvirus vaccinations (e.g. zostavax) and herpesvirus treatments (e.g. with antiviral agents such as acyclovir).

Comparators: Comparators will vary by study design and will include subjects randomised not to receive an antiviral intervention or vaccination (RCTs), people unexposed to herpesvirus infections (cohort studies and case control studies) and person time unexposed to herpesviruses (self-controlled case series or case crossover studies).

Outcome: Primary outcomes are dementia (all types), diagnosed either clinically, with or without neuroimaging or by histopathology, and MCI, characterised clinically. Where possible, dementia will be further categorised by type, e.g. AD, vascular dementia, fronto-temporal dementia and its sub-categories, Lewy body dementia, and other rare subtypes including Huntington's disease and prion diseases.

Literature searches

We will search the Cochrane Library, Embase, Global Health, Medline, PsychInfo, Scopus and Web of Science from dates of inception to January 2017. We will also search clinical trials registers and grey literature including ClinicalTrials.gov, the New York Academy of Medicine Grey Literature Report (www.greylit.org), the Electronic Theses Online Service through the British Library

For peer review only - http://bmjopen.bmj.com/site/about/guidelines.xhtml

(http://ethos.bl.uk) and the ISI Conference Proceedings Citation Index (http://isknowledge.com). We will use medical subject heading (MeSH) terms and keyword searches (in the title and abstract) to capture studies of infection with, vaccination against or antiviral treatment of any of the eight human herpesviruses (HSV1, HSV2, VZV, EBV, cytomegalovirus (CMV), human herpesvirus 6 (HHV6), HHV7, HHV8) AND dementia (including subtypes) or MCI. This strategy will be supplemented by reviewing reference lists of eligible articles and relevant reviews. The search strategy has been developed in Medline and will be translated for use in other databases (Appendix 1).

Study records

Data management: Search results will be uploaded into an EndNote database (V7.5) and de-duplicated.

Selection process: All titles and abstracts will be scanned for eligibility by two researchers in parallel. The full texts of all articles that potentially meet inclusion criteria will then be obtained and again reviewed in parallel. We will note reasons for rejection of articles at this stage according to a hierarchical list (ineligible study design, wrong exposure, wrong outcome, insufficient information to calculate an effect estimate). Any discrepancies will be discussed between reviewers and consultation with a third reviewer will be carried out where necessary.

Data collection process: The data items listed below will be incorporated into a pilot data extraction table. Parallel data extraction will be carried out for the first three included studies by two reviewers and changes to the extraction table made as required. Any discrepancies will be discussed, and resolved through consultation with a third reviewer if necessary. Data will be extracted for each remaining study by a single reviewer. We will also consider contacting corresponding authors of published studies to obtain any further information needed using a standardised email template.

Data items

We will design a data extraction table to collect information on the following domains:

For peer review only - http://bmjopen.bmj.com/site/about/guidelines.xhtml

For peer review only

Population: characteristics of the study population (e.g. sex and age distribution, ethnicity, immune status, APOE4 status), recruitment and sampling methods, inclusion/exclusion criteria;

Exposure: exposure status definition and identification, number of exposed subjects, any exclusions;

Comparators: identification and definition of unexposed individuals, number of unexposed subjects, any exclusions;

Outcomes: definition and identification of primary (dementia or MCI) and secondary outcomes (dementia subtypes), number of subjects, any exclusions;

Study characteristics: authors, publication year, setting/source of participants, design, period of study, length of follow-up time (if relevant), aims and objectives.

We will record both unadjusted and fully adjusted effect estimates for the association between each of the human herpesviruses and risk or rate of dementia or MCI. Details of the confounders measured and adjusted for will be noted. Results of any additional stratified analyses will also be recorded, e.g. on virus effects in different population subgroups or on dementia subtypes, including categories of severity.

Outcomes and prioritization

The primary outcomes are dementia (all types) and MCI. Where possible, the neurodegenerative condition leading to the syndromes of dementia or MCI will be recorded, e.g. AD, vascular dementia, mixed AD and vascular dementia, fronto-temporal dementia and its sub-categories, Lewy body dementia, and other rare subtypes including Huntington's disease and prion diseases. A priori, we anticipate that most studies will report on AD. We have, however, opted for a broader primary outcome definition: excluding studies that do not record dementia type will limit the statistical power to detect an effect yet identifying a precise dementia aetiology is challenging, especially when histopathological diagnosis is not available. We will prioritise studies in which herpesvirus status was

For peer review only - http://bmjopen.bmj.com/site/about/guidelines.xhtml

For peer review only

ascertained prior to the occurrence of an outcome event when assessing study quality. These are likely to be longitudinal prospective studies that generate incidence rate ratios or hazard ratios, but we will also consider estimates of odds ratios from case control studies.

Risk of bias in individual studies

We will assess the risk of bias in individual studies in line with the Cochrane Collaboration approach for both randomised and non-randomised studies[17,18]. For RCTs this will include consideration of the effects of selection bias, performance bias, detection bias, attrition bias and reporting bias. For observational studies we will consider relevant domains including selection of participants, measurement of variables, control for confounding and missing data. For each study, each component will be assigned a risk of bias category 'high risk', 'low risk' or 'unclear risk' and a summary risk of bias table produced. Two reviewers will assign risk of bias categories in parallel for the first three studies and, as before, any discrepancies that cannot be resolved will be discussed with a third reviewer. One reviewer will then assign risk of bias categories for the remaining studies.

Data synthesis

We will conduct a narrative synthesis in which evidence is grouped by exposure and outcome definitions and we will additionally summarise the results in tables. We will assess whether there are sufficient data to investigate the effect of herpesviruses on population subgroups or subtypes of dementia. We will investigate whether vaccination to prevent herpesvirus infection or reactivation e.g. using chickenpox vaccine or herpes zoster vaccine mitigates the risk of dementia. We will also present any evidence for a modulating effect of antiviral treatment against herpesviruses on dementia.

We will consider performing a meta-analysis if there are sufficient numbers of studies with the same design and sufficiently homogeneous populations, exposures and outcomes to calculate pooled

For peer review only - http://bmjopen.bmj.com/site/about/guidelines.xhtml

effect estimates. For case control studies we will present pooled odds ratios, for cohort studies, pooled rate or hazard ratios and for case only designs, pooled incidence ratios will be calculated. The choice of fixed or random effects models will be guided by the level of statistical heterogeneity. We will consider the magnitude and direction of effects when interpreting I^2 values, but will generally take an I^2 value of >25% to represent moderate or substantial heterogeneity[19,20]. Sources of heterogeneity will be explored by considering the use of meta-regression to compare summary estimates from different study-level characteristics, such as age of the study population, study design and dementia or MCI diagnosis. In sensitivity analysis, the most biased studies (those with more than one domain classified as high risk of bias) will be excluded. If there are sufficient numbers of studies, we will investigate the risk of publication bias using a funnel plot. All of the statistical analysis will be performed using STATA version 14.0.

Cumulative evidence

The quality of cumulative evidence across studies for an association of each herpesvirus with each outcome will then be assessed using selected GRADE criteria that are relevant to the included studies[21]. As well as risk of bias these include inconsistency, indirectness, imprecision, publication bias and any additional domains deemed appropriate to categorise the strength of evidence as 'high', 'moderate' or 'low/very low'.

ETHICS AND DISSEMINATION

Ethical approval was not required as this study is a systematic review of previously published studies. The protocol was registered with the International Prospective Register of Systematic Reviews (PROSPERO) on 7 January 2017 (Registration number: CRD42017054684). Any future amendments will be documented on the PROSPERO website. Results will be submitted for publication in a peer-reviewed journal and presented at national and international conferences.

For peer review only - http://bmjopen.bmj.com/site/about/guidelines.xhtml

Contributors

CWG conceived and designed the study, drafted and revised the protocol following author comments; HF contributed to the design of the study and revised the paper critically; JB contributed to the conception and design of the study and revised the paper critically; ACH contributed to the conception and design of the study and revised the paper critically; AM contributed to the design of the study and revised the paper critically; BHR contributed to the design of the study and revised the paper critically; MR contributed to the design of the study and revised the paper critically; SLT contributed to the design of the study and revised the paper critically; LS contributed to the conception and design of the study and revised the paper critically. All authors approved the final version of the protocol.

Funding

This work was supported by a Wellcome Intermediate Clinical Fellowship awarded to CW-G (201440/Z/16/Z). LS is supported by a Senior Clinical Fellowship from Wellcome (098504/Z/12/Z). JB receives funding from the NIHR UCL/UCLH BRC. The funder had no direct role in developing the protocol.

Competing interests

All authors report no conflicts of interest

References

1. Office for National Statistics. Deaths registered in England and Wales (Series DR): 2015. Release date 14 Nov 2016. Available from: https://www.ons.gov.uk/peoplepopulationandcommunity/birthsdeathsandmarriages/deaths/bulletins/deathsregisteredinenglandandwalesseriesdr/2015. Last accessed 13 Feb 2017.

2. Prince M, Wimo A, Guerchet M, Ali G-C, Wu Y-T, Prina M. World Alzheimer Report 2015. The Global Impact of Dementia: An Analysis of Prevalence, Incidence, Cost and Trends. [Internet]. London, UK: Alzheimer's Disease International. Aug 2015. Available from: https://www.alz.co.uk/research/WorldAlzheimerReport2015.pdf Last accessed 13 Feb 2017.

For peer review only - http://bmjopen.bmj.com/site/about/guidelines.xhtml

For peer review only

3. Prince M, Ali G-C, Guerchet M, Prina AM, Albanese E, Wu Y-T. Recent global trends in the prevalence and incidence of dementia, and survival with dementia. Alzheimers Res Ther. 2016; 8(1):23.

4. Norton S, Matthews FE, Barnes DE, Yaffe K, Brayne C. Potential for primary prevention of Alzheimer's disease: an analysis of population-based data. Lancet Neurol. 2014; 13(8):788–794.

5. Agostini S, Clerici M, Mancuso R. How plausible is a link between HSV-1 infection and Alzheimer's disease? Expert Rev Anti Infect Ther. 2014;12(3):275–8.

6. Itzhaki RF, Lathe R, Balin BJ, Ball MJ, Bearer EL, Braak H, et al. Microbes and Alzheimer's Disease. J Alzheimers Dis. 2016; 51(4):979–84.

7. Whitley RJ. Herpesviruses. In: Baron S, editor. Medical Microbiology. 4th ed. Galveston (TX): University of Texas Medical Branch at Galveston; 1996. Available from: http://www.ncbi.nlm.nih.gov/books/NBK8157/ Last accessed 13 Feb 2017.

8. Alvarez G, Aldudo J, Alonso M, Santana S, Valdivieso F. Herpes simplex virus type 1 induces nuclear accumulation of hyperphosphorylated tau in neuronal cells. J Neurosci Res. 2012; 90(5):1020–9.

9. Wozniak MA, Itzhaki RF, Shipley SJ, Dobson CB. Herpes simplex virus infection causes cellular β-amyloid accumulation and secretase upregulation. Neurosci Lett. 2007; 429(2–3):95–100.

10. De Chiara G, Marcocci ME, Civitelli L, Argnani R, Piacentini R, Ripoli C, et al. APP Processing Induced by Herpes Simplex Virus Type 1 (HSV-1) Yields Several APP Fragments in Human and Rat Neuronal Cells. Blaho JA, editor. PLoS ONE. 2010; 5(11): e13989.

11. Wozniak MA, Frost AL, Preston CM, Itzhaki RF. Antivirals reduce the formation of key Alzheimer's disease molecules in cell cultures acutely infected with herpes simplex virus type 1. PloS One. 2011; 6(10): e25152.

12. Baringer JR, Pisani P. Herpes simplex virus genomes in human nervous system tissue analyzed by polymerase chain reaction. Ann Neurol. 1994; 36(6): 823–9.

13. Yao H-W, Ling P, Chen S-H, Tung Y-Y, Chen S-H. Factors affecting herpes simplex virus reactivation from the explanted mouse brain. Virology. 2012; 433(1): 116–23.

14. Chen S-H, Yao H-W, Huang W-Y, Hsu K-S, Lei H-Y, Shiau A-L, et al. Efficient Reactivation of Latent Herpes Simplex Virus from Mouse Central Nervous System Tissues. J Virol. 2006; 80(24): 12387–92.

15. Steel AJ, Eslick GD. Herpes Viruses Increase the Risk of Alzheimer's Disease: A Meta-Analysis. J Alzheimers Dis. 2015; 47(2): 351–64.

16. Moher D, Shamseer L, Clarke M, Ghersi D, Liberati A, Petticrew M, et al. Preferred reporting items for systematic review and meta-analysis protocols (PRISMA-P) 2015 statement. Syst Rev. 2015; 4(1): 1.

17. Higgins JPT, Altman DG, Gøtzsche PC, Jüni P, Moher D, Oxman AD, et al. The Cochrane Collaboration's tool for assessing risk of bias in randomised trials. BMJ. 2011; 343: d5928.

For peer review only - http://bmjopen.bmj.com/site/about/guidelines.xhtml

18. Sterne JA, Hernán MA, Reeves BC, Savović J, Berkman ND, Viswanathan M, et al. ROBINS-I: a tool for assessing risk of bias in non-randomised studies of interventions. BMJ. 2016; 355: i4919.

19. Higgins JPT, Green S. Section 9.5.2 Identifying and measuring heterogeneity in Cochrane handbook for systematic reviews of interventions. Version 5.1.0. Mar 2011. Available from: http://handbook.cochrane.org/chapter_9/9_5_2_identifying_and_measuring_heterogeneity.htm Last accessed 04 May 2017.

20. Riley RD, Higgins JPT, Deeks JJ. Interpretation of random effects meta-analyses. *BMJ* 2011; 342:d549

21. Atkins D, Best D, Briss PA, Eccles M, Falck-Ytter Y, Flottorp S, et al. Grading quality of evidence and strength of recommendations. BMJ. 2004; 328(7454): 1490.

For peer review only - http://bmjopen.bmj.com/site/about/guidelines.xhtml

For peer review only

1 exp Herpes simplex
2 exp Herpes simplex virus vaccines
3 exp encephalitis, herpes simplex
4 exp Herpesvirus 1, Human
5 cold sore$.ti,ab.
6 exp Herpesvirus 2, Human
7 (genit$ herpes$ or genit$ sores).ti,ab.
8 exp Chickenpox
9 exp Chickenpox vaccine
10 exp Herpes zoster
11 exp Neuralgia, postherpetic
12 exp Herpesvirus 3, Human
13 exp Encephalitis, varicella zoster
14 (varicella or chickenpox or chicken pox or shingles or VZV or zoster).ti,ab.
15 exp Cytomegalovirus
16 exp Cytomegalovirus vaccines
17 exp Cytomegalovirus infections
18 (CMV or cytomegalovirus).ti,ab.
19 exp Herpesvirus 6, Human
20 Roseolovirus Infections/
21 Exanthema Subitum/
22 (B lymphotropic virus$ or roseola or sixth disease or exanthema subitum or exanthem criticum or Roseolovirus or pseudorubella or three?day fever).ti,ab.
23 exp Herpesvirus 7, Human
24 exp Epstein-Barr virus infections
25 exp Epstein-Barr virus
26 exp Herpesvirus 4, Human
27 (EBV or epstein-barr or burkitt adj5 lymphoma$ or glandular fever or infectious mono$ or mononucleosis or hair$ leukoplak$ or OHL).ti,ab.
28 exp Herpesvirus 8, Human
29 Sarcoma, Kaposi/
30 Lymphoma, Primary Effusion/
31 (kaposi$ sarcoma$ or Primary effusion adj2 lymphoma$ or body cavity adj2 lymphoma$).ti,ab.
32 ((HHV adj1 ("1" or "2" or "3" or "4" or "5" or "6" or "7" or "8")) or (HHV?1 or HHV?2 or HHV?3 or HHV?4 or HHV?5 or HHV?6 or HHV?7 or HHV?8)).ti,ab.
33 (HSV?1 or HSV 1 or HSV?2 or HSV 2).ti,ab.
34 herpes$.ti, ab.
35 exp acyclovir
36 ganciclovir/ or foscarnet/ or Idoxuridine/ or Trifluridine/
37 (ac?clovir or Zovirax or valac?clovir or valtrex or famc?clovir or famvir or penc?clovir or ganc?clovir or cidofovir or foscarnet$ or valganc?clovir or lubocavir or brivudin or Docosanol or Sorivudine or Idoxuridine or Trifluridine).ti,ab

38 or 1/37
39 exp dementia/
40 exp mild cognitive impairment/
41 (Alzheimer$ or dementia or Kluver-Bucy or huntington$).ti, ab.
42 (lewy$ adj2 bod$).ti, ab.
43 (Pick$ disease$ AND brain$).ti, ab.
44 ((memory or cognit$ or mental) adj5 (los$ or impair$ or deficit or problem or damage or declin$ or deteriorat$ or degenerat$ or diminish$)).ti, ab.
45 Supranuclear Palsy, Progressive/
46 (supra?nuclear pals$ or supra nuclear pals$ or PSP).ti, ab.
47 prion diseases/ or creutzfeldt-jakob syndrome/ or gerstmann-straussler-scheinker disease/ or insomnia, fatal familial/ or kuru/
48 (Prion$ disease$ or fatal familial insomnia or FFI or Gertsmann-Straussler-Scheinker syndrome or GSS or kuru or variab$ protease-sensitive prionopathy or VPSPr or transmissible spongiform encephalopath$ or TSE or Creutzfeld-Jacob$ or JCD or CJD).ti, ab.
49 or 39/49
50 38 and 51

For peer review only

PRISMA-P (Preferred Reporting Items for Systematic review and Meta-Analysis Protocols) 2015 checklist: recommended items to address in a systematic review protocol*

Section and topic	Item No	Checklist item		
**ADMINISTRATIVE INFORMATION**				
Title:				
Identification	1a	Identify the report as a protocol of a systematic review	✓	P1
Update	1b	If the protocol is for an update of a previous systematic review, identify as such	-	-
Registration	2	If registered, provide the name of the registry (such as PROSPERO) and registration number	✓	P2
Authors:				
Contact	3a	Provide name, institutional affiliation, e-mail address of all protocol authors; provide physical mailing address of corresponding author	✓	P1
Contributions	3b	Describe contributions of protocol authors and identify the guarantor of the review	✓	P10/11
Amendments	4	If the protocol represents an amendment of a previously completed or published protocol, identify as such and list changes; otherwise, state plan for documenting important protocol amendments	-	-
Support:				
Sources	5a	Indicate sources of financial or other support for the review	✓	P11
Sponsor	5b	Provide name for the review funder and/or sponsor	✓	P11
Role of sponsor or funder	5c	Describe roles of funder(s), sponsor(s), and/or institution(s), if any, in developing the protocol	✓	P11
**INTRODUCTION**				
Rationale	6	Describe the rationale for the review in the context of what is already known	✓	P4/5
Objectives	7	Provide an explicit statement of the question(s) the review will address with reference to participants, interventions, comparators, and outcomes (PICO)	✓	P5
**METHODS**				
Eligibility criteria	8	Specify the study characteristics (such as PICO, study design, setting, time frame) and report characteristics (such as years considered, language, publication status) to be used as criteria for eligibility for the review	✓	P6
Information sources	9	Describe all intended information sources (such as electronic databases, contact with study authors, trial registers or other grey literature sources) with planned dates of coverage	✓	P6/7

For peer review only

Search strategy	10	Present draft of search strategy to be used for at least one electronic database, including planned limits, such that it could be repeated	✓	P7 (and in an appendices)
Study records:				
Data management	11a	Describe the mechanism(s) that will be used to manage records and data throughout the review	✓	P7
Selection process	11b	State the process that will be used for selecting studies (such as two independent reviewers) through each phase of the review (that is, screening, eligibility and inclusion in meta-analysis)	✓	P7
Data collection process	11c	Describe planned method of extracting data from reports (such as piloting forms, done independently, in duplicate), any processes for obtaining and confirming data from investigators	✓	P7
Data items	12	List and define all variables for which data will be sought (such as PICO items, funding sources), any pre-planned data assumptions and simplifications	✓	P7/8
Outcomes and prioritization	13	List and define all outcomes for which data will be sought, including prioritization of main and additional outcomes, with rationale	✓	P8
Risk of bias in individual studies	14	Describe anticipated methods for assessing risk of bias of individual studies, including whether this will be done at the outcome or study level, or both; state how this information will be used in data synthesis	✓	P9
Data synthesis	15a	Describe criteria under which study data will be quantitatively synthesised	✓	P9/10
	15b	If data are appropriate for quantitative synthesis, describe planned summary measures, methods of handling data and methods of combining data from studies, including any planned exploration of consistency (such as I^2, Kendall's τ)	✓	P9/10
	15c	Describe any proposed additional analyses (such as sensitivity or subgroup analyses, meta-regression)	✓	P9/10
	15d	If quantitative synthesis is not appropriate, describe the type of summary planned	✓	P9/10
Meta-bias(es)	16	Specify any planned assessment of meta-bias(es) (such as publication bias across studies, selective reporting within studies)	✓	P9/10
Confidence in cumulative evidence	17	Describe how the strength of the body of evidence will be assessed (such as GRADE)	✓	P10

*** It is strongly recommended that this checklist be read in conjunction with the PRISMA-P Explanation and Elaboration (cite when available) for important clarification on the items. Amendments to a review protocol should be tracked and dated.** **The copyright for PRISMA-P (including checklist) is held by the PRISMA-P Group and is distributed under a Creative Commons Attribution Licence 4.0.**

From: Shamseer L, Moher D, Clarke M, Ghersi D, Liberati A, Petticrew M, Shekelle P, Stewart L, PRISMA-P Group. Preferred reporting items for systematic review and meta-analysis protocols (PRISMA-P) 2015: elaboration and explanation. BMJ. 2015 Jan 2;349(jan02 1):g7647.

For peer review only
